# Selective Area Epitaxy of GaN Nanowires on Si Substrates Using Microsphere Lithography: Experiment and Theory

**DOI:** 10.3390/nano12142341

**Published:** 2022-07-08

**Authors:** Vladislav O. Gridchin, Liliia N. Dvoretckaia, Konstantin P. Kotlyar, Rodion R. Reznik, Alesya V. Parfeneva, Anna S. Dragunova, Natalia V. Kryzhanovskaya, Vladimir G. Dubrovskii, George E. Cirlin

**Affiliations:** 1 Department of Physics, Alferov University, Khlopina 8/3, 194021 St. Petersburg, Russia; gridchinvo@gmail.com (V.O.G.); liliyabutler@gmail.com (L.N.D.); moment92@mail.ru (R.R.R.); 2 Faculty of Physics, St. Petersburg State University, Universitetskaya Embankment 13B, 199034 St. Petersburg, Russia; konstantin21kt@gmail.com; 3 Institute for Analytical Instrumentation RAS, Rizhsky 26, 190103 St. Petersburg, Russia; 4 Department of Physics, ITMO University, Kronverkskiy Pr. 49, 197101 St. Petersburg, Russia; 5 Ioffe Institute, Polytechnicheskaya 26, 194021 St. Petersburg, Russia; cheal@mail.ioffe.ru; 6 Department of Physics, Higher School of Economics, Kantemirovskaya 3/1 A, 194100 St. Petersburg, Russia; adragunova@hse.ru (A.S.D.); nkryzhanovskaya@hse.ru (N.V.K.)

**Keywords:** GaN nanowires, selective area growth, molecular beam epitaxy, modeling, optical properties

## Abstract

GaN nanowires were grown using selective area plasma-assisted molecular beam epitaxy on SiO_x_/Si(111) substrates patterned with microsphere lithography. For the first time, the temperature–Ga/N_2_ flux ratio map was established for selective area epitaxy of GaN nanowires. It is shown that the growth selectivity for GaN nanowires without any parasitic growth on a silica mask can be obtained in a relatively narrow range of substrate temperatures and Ga/N_2_ flux ratios. A model was developed that explains the selective growth range, which appeared to be highly sensitive to the growth temperature and Ga flux, as well as to the radius and pitch of the patterned pinholes. High crystal quality in the GaN nanowires was confirmed through low-temperature photoluminescence measurements.

## 1. Introduction

GaN nanowires (NWs) have recently attracted much attention as building blocks for a wide range of optoelectronic devices, including photonic-crystal lasers with multi-color emission in the visible range and single-photon sources operating at room temperature [1,2,3,4]. Epitaxial growth of GaN NWs on lattice-mismatched Si substrates makes it possible to obtain exceptional crystal quality in NWs [5,6] while providing an opportunity to use Si as the ohmic contact for GaN [7] and develop III-N-based UV and visible LEDs integrated with a Si electronic platform [8,9]. Single- and entangled-photon sources based on III-N materials on silicon can be used as building blocks in quantum networks and hold promise for applications in quantum informatics and telecommunications [1,10,11,12].

The statistical nature of the NW nucleation and growth process leads to inhomogeneous size distributions for the GaN NWs, in terms of both diameters and lengths, and inhibits the reproducibility of NW growth. To fabricate NW-based light emitting structures, it is crucial to maintain the necessary control over the NW spacing, diameter and length and improve the size uniformity across large surface areas. All these factors directly affect the electrical transport [13] and light emission properties of the GaN NW ensembles [14]. Furthermore, fabrication of nonlinear photonic crystals based on highly ordered GaN NWs is crucial for the emission of entangled photons [15,16].

One way to grow uniform arrays of GaN NWs is selective area epitaxy (SAE) on patterned substrates. Most commonly, patterned pinholes in a mask (for example, SiN_x_ or SiO_x_) are formed using electron beam lithography [17,18,19,20,21,22,23]. As regards growth studies, Gotschke et al. [17] investigated the influence of Ga adatom diffusion on the SAE of GaN NWs on SiO_x_/AlN/Si(111) with different mask configurations. Kruse et al. studied the influence of AlN interlayers between the mask and Si substrate on the structural properties of SAE-grown GaN NWs [18]. Schuster et al. carried out a comprehensive study of the influence of temperature, III/V flux ratio, period and pinhole diameter using different substrates on the GaN NW morphology [21]. To the best of our knowledge, the influence of the III/V flux ratio on the SAE of GaN NWs was only investigated by changing the N_2_ flux at a constant temperature and Ga flux [21].

In this work, we studied the effect of temperature and the Ga/N_2_ flux ratio with different Ga fluxes and a fixed N_2_ flux on the SAE of GaN NWs on patterned SiO_x_/Si(111) substrates using plasma-assisted molecular beam epitaxy (PA-MBE). Microsphere lithography was employed to form the pinholes in the SiO_x_ mask layer. This patterning method provides sub-micrometer lateral resolution in a versatile, scalable and cost-effective way [24]. The pattern geometry can easily be tuned by changing the diameter of the microspheres, while a spin-coating process enables processing of large-area substrates [25,26]. Pre-deposition of interlayers, such as AlN, was intentionally excluded for achieving the direct contact between Si and GaN. A growth diagram separating three domains— SAE in the absence of any parasitic NWs on the mask surface, parasitic growth and no growth—was obtained as a function of the temperature and Ga/N_2_ flux ratio. A dedicated model was developed that explained the SAE growth map and showed that the growth selectivity was influenced not only by the temperature and Ga flux but also by the geometrical parameters of the template. As a result, the very possibility of SAE growth of GaN NWs on patterned SiO_x_/Si(111) substrates critically depends on the correct choice of temperature and Ga/N_2_ flux ratio for pinholes of a given size and pitch.

## 2. Experimental

**Figure 1:**
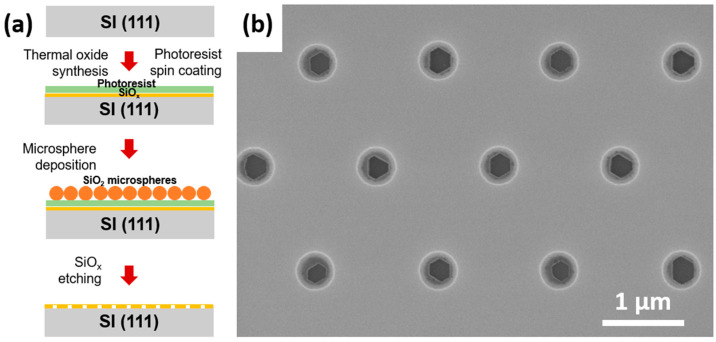
(**a**) Illustration of SiO_x_/Si(111) substrate patterning process, including thermal oxide synthesis, spin-coating of photoresist, microsphere deposition, UV exposure of photoresist and SiO_x_ etching. (**b**) SEM image of patterned pinholes in SiO_x_ mask on a Si(111) substrate.

Figure 1 illustrates the surface patterning process and a plan-view scanning electron microscopy (SEM) image of the patterned SiO_x_/Si(111) substrate. Si(111) substrates (1 × 10^16^ cm^−3^ electron concentration) were thermally oxidized to form a 60 nm thin SiO_x_ layer. Microsphere photolithography and plasma etching were employed to pattern the oxide layer into a regular array of pinholes. First, dense arrays of SiO_2_ microspheres were spin-coated on the photoresist layer covering the Si(111) substrate. The optimal parameters of the microsphere deposition can be found in our previous work [25]. Second, the photoresist was exposed to 365 nm UV radiation. Every microsphere acted as a lens focusing the UV light onto the optical jet underneath [27]. During the development of the photoresist, the microspheres were spined off the substrate. The patterned photoresist layer served as a template for further inductively coupled SF6 etching of the oxide. Finally, the photoresist layer was removed to form the patterned SiO_x_/Si(111) substrates with ordered arrays of submicron-sized pinholes. This approach allows one to pattern large-area Si substrates up to several inches in diameter. Typical scanning electron microscopy (SEM) images of the substrates are shown in Figure 1b.

We used SiO_2_ microspheres with 1.8 µm diameters, which resulted in a large pitch for the pinhole arrays and, consequently, large separation between the NWs. The low NW surface density eliminated the possible negative effects on the size uniformity of the GaN NW, including the competition of the neighboring NWs for Ga diffusion flux and the shadowing effect in the directional MBE method. 

The GaN NWs were grown using PA-MBE on pre-patterned one-quarter 2 inch SiO_x_/Si(111) substrates in a Riber Compact 12 MBE equipped with a Ga effusion cell and N_2_ plasma source. Prior to growth, the substrates were heated up to a temperature of 915 °C and annealed for 20 min to remove the native oxide. This process was controlled in situ with reflection high-energy electron diffraction. Twenty minutes of annealing at this temperature enabled native oxide desorption without any destruction of the SiO_x_ mask. Then, the substrate temperature was reduced to an NW growth temperature, the nitrogen plasma source was ignited and the Ga shutter opened. The N_2_ flow was fixed at 0.4 sccm, and the nitrogen plasma source power was fixed at 450 W. We then carried out the series of experiments with different beam equivalent pressures (BEPs) for the Ga flux at a constant substrate temperature. Next, we increased the substrate temperature in steps of 5 °C and carried out MBE growth at the same Ga BEPs as before. Overall, the growth experiments were conducted within a rectangular range in the temperature–Ga BEP plane from 820 to 850 °C, for the temperature, and from 1 × 10^−7^ Torr to 5 × 10^−^^7^ Torr, for the Ga BEP. Several growth experiments were carried out using a specially designed substrate holder, which provided a high temperature gradient across the substrate surface [28]. For this holder, our measurements using an OPTRIS Compact CT Laser 3MH1 pyrometer gave a temperature difference of 25 °C between the center and the edge of the one-quarter 2-inch substrate for a temperature of 840 °C at the center. This difference was carefully accounted for when analyzing the temperature dependence of the NW morphology. 

The morphology of the samples was studied with scanning electron microscopy (SEM) using a Supra 25 Zeiss SEM. The photoluminescence (PL) measurements were recorded at room temperature and 6 K using a He–Cd metal-vapor laser with a wavelength of 325 nm at 6.5 mW. The laser spot diameter was 100 µm. The PL signal was detected using a DK480 Spectral Products monochromator and a single-channel Si detector in synchronous detection mode (SRS 510, Stanford Research Systems, Sunnyvale, CA, USA).

## 3. Results and Discussion

### 3.1. Influence of the MBE Growth Conditions on the NW Morphology 

**Figure 2:**
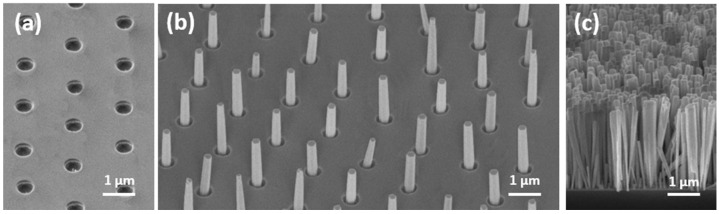
Isometric SEM images of GaN NWs grown at 830 °C and different Ga BEPs of (**a**) 2 × 10^−7^ Torr, (**b**) 3 × 10^−7^ Torr and (**c**) 5 × 10^−7^ Torr.

**Figure 3:**
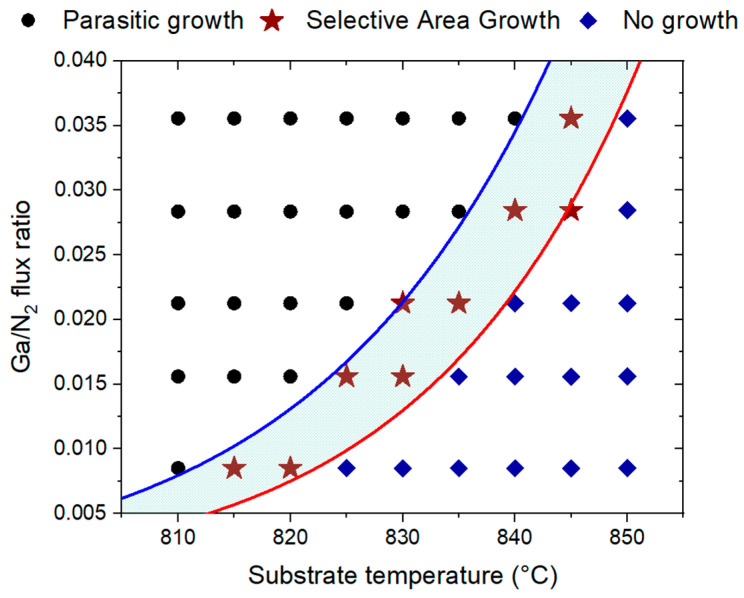
Temperature–Ga/N_2_ flux-ratio growth diagram showing the data points corresponding to parasitic-growth, SAE and no-growth conditions for GaN NWs on patterned SiO_x_/Si(111) substrates. The curves separating the three domains are the fits obtained with the model.

Figure 2 shows isometric SEM images of GaN NWs grown at a substrate temperature of 830 °C and different Ga BEPs. These SEM images clearly show the three possible growth modes: no growth of NWs at a low Ga BEP of 2 × 10^−7^ Torr in Figure 2a (“no growth” in what follows), true SAE of NWs without any parasitic NW growth on the mask surface in Figure 2b (“SAE growth”) and uncontrolled growth where NWs form in the pinholes and on the SiO_x_ mask itself in Figure 2c (“parasitic growth”). The uniformity of the GaN NWs shown in Figure 2b was within a range of ±17 for the radius and ±13% for the length. These values were higher than for GaN NWs grown on patterned diamond substrates using e-beam lithography [21] but can be further improved by increasing the Si smoothness in the pinholes. For each Ga BEP and temperature, one of the three growth modes was determined. Figure 3 shows the resulting growth map in terms of temperature and Ga/N_2_ flux ratio. The SAE growth region on the map is relatively narrow and restricted by the no-growth region below and the parasitic-growth region above the SAE zone. For a given temperature, the Ga/N_2_ flux ratio should be neither too high nor too low to ensure growth selectivity, and it should be accurate within the order of only 0.005. Lower Ga/N_2_ flux ratios corresponded to no growth, while higher Ga/N_2_ flux ratios led to parasitic NW growth on the mask surface. For a given Ga/N_2_ flux ratio, the temperature should be within an optimal range, and it should be accurate within the order of only 10 °C. Lower temperatures yielded parasitic growth, while higher temperatures resulted in no growth. 

### 3.2. Model 

For self-catalyzed nucleation and growth of III-V NWs, it is well-known that group III flux should be neither too high nor too low in order to improve the yield of vertical NWs in the pinholes at a given temperature (see, for example, Ref [29] and references therein). The nucleation process for the SAE III-V NWs is controlled by surface diffusion of group III adatoms from the mask to the pinholes [29]. It is not guaranteed, however, that the diffusion flux will be directed into the pinholes rather than in the opposite direction, and it may also be cancelled under steady-state conditions [30]. For self-induced nucleation of GaN NWs, the incubation time before nucleation may reach several hours [31,32], as most Ga atoms desorb from the surface at the high temperatures typically employed for MBE growth. Therefore, the diffusion flux of Ga atoms on the substrate surface is very low compared to their impingement and desorption rates. This was also expected to be the case for our growth conditions. 

To understand the trends shown in Figure 3 and the separation between the SAE, no-growth, and parasitic-growth domains, we established the following semi-quantitative model. The true SAE of GaN NWs requires that (i) NWs nucleate in the pinholes and (ii) no NWs emerge on the mask surface between the pinholes. In terms of the nucleation rates of GaN NWs on the Si(111) surface within the openings, J, and on the SiO_x_ mask surface, $\bar{J}$, these two requirements can be quantified as:

(1) $$J>\frac{1}{\pi R^{2}\tau_{3}},\;\bar{J}<\frac{1}{\left(S-\pi R^{2}\right){\bar{\tau }}_{3}}.$$

The first inequality means that GaN NWs nucleate inside the pinholes with surface area $\pi R^{2}$ (with R as the pinhole radius) during the mean stay time of the Ga atoms on the Si(111) surface before desorption $\tau_{3}$. The second inequality ensures the absence of parasitic nucleation on the mask surface of area $S-\pi R^{2}$ (with S as the surface area per pinhole, and $S=P^{2}$ for a square array of pinholes with pitch P) around the pinhole during the mean stay time of the Ga atoms on the SiO_x_ ${\bar{\tau }}_{3}$. In the irreversible growth model [31], the meeting of any two Ga (labelled “3”) and N (labelled “5”) adatoms on the Si(111) surface leads to NW nucleation. Hence, the nucleation rate of GaN NWs is given by:

(2) $$J=D_{3}n_{3}n_{5}.$$

Here, $D_{3}$ is the diffusion coefficient of Ga adatoms on Si(111), $n_{3}$ is their surface concentration and $n_{5}$ is the surface concentration of N adatoms.

All Ga and N atoms arriving onto the Si(111) surface inside the pinhole at the rates $I_{3}$ and $I_{5}$, respectively, either desorb or contribute to NW nucleation. Therefore,

(3) $$I_{3}=\frac{n_{3}}{\tau_{3}}+D_{3}n_{3}n_{5},\;I_{5}=2D_{5}n_{5}^{2}+D_{3}n_{3}n_{5}≅2D_{5}n_{5}^{2},$$

where we take into account that N desorbs in the form of N_2_ molecules made of two N atoms that meet due to surface diffusion with the diffusivity $D_{5}$. Equation (3) neglects the possible diffusion of Ga adatoms into or from the pinhole [29,30,31] in the first approximation. This requires relatively low values for the surface diffusion of Ga adatoms compared to their arrival and desorption rates, which seems reasonable at the high growth temperatures employed here and is supported by the results from previous studies [31,32]. Assuming that the GaN nucleation rate is much lower that the desorption rate of N atoms, which corresponds to the approximate Equation (3) for N atoms, we can express the unknown $n_{3}$ and $n_{5}$ through the fluxes and diffusion coefficients. Using the expressions obtained in Equation (2), Equation (1) for J becomes:

(4) $$I_{3}>\frac{1}{\pi R^{2}\tau_{3}}\frac{1+D_{3}\tau_{3}\sqrt{I_{5}/2D_{5}}}{D_{3}\tau_{3}\sqrt{I_{5}/2D_{5}}}.$$

Repeating the same considerations for Ga adatoms on the SiO_x_ surface, Equation (1) for $\bar{J}$ gives:

(5) $$I_{3}<\frac{1}{\left(S-\pi R^{2}\right){\bar{\tau }}_{3}}\frac{1+{\bar{D}}_{3}{\bar{\tau }}_{3}\sqrt{I_{5}/2{\bar{D}}_{5}}}{{\bar{D}}_{3}{\bar{\tau }}_{3}\sqrt{I_{5}/2{\bar{D}}_{5}}},$$

where ${\bar{D}}_{3}$ and ${\bar{D}}_{5}$ are the diffusion coefficients of Ga and N adatoms on the mask surface. 

To access the temperature dependence of the conditions for pure SAE growth given by Equations (4) and (5), we used the standard Arrhenius expressions for the diffusion coefficients ($D_{3}=l_{D}^{2}\nu_{diff}^{\left(3\right)}exp(-E_{diff}^{\left(3\right)}/k_{B}T)$, $D_{5}=l_{D}^{2}\nu_{diff}^{\left(5\right)}exp(-E_{diff}^{\left(5\right)}/k_{B}T)$) and for the mean stay time of Ga on Si(111) before desorption ($\tau_{3}=exp\left(E_{des}^{\left(3\right)}/k_{B}T\right)/\nu_{des}^{\left(3\right)}$) [32], and similar expressions were used for SiO_x_. Here, $l_{D}$ is the length of one diffusion “jump” on a surface (a value in the order of lattice spacing), $\nu_{diff}^{\left(3\right)}$ and $\nu_{diff}^{\left(5\right)}$ are the characteristic vibration frequencies in the Arrhenius temperature dependences of the diffusion coefficients for Ga and N adatoms, respectively, $E_{diff}^{\left(3\right)}$ and $E_{diff}^{\left(5\right)}$ are the activation energies for the surface diffusion of Ga and N adatoms, respectively, $\nu_{des}^{\left(3\right)}$ is the characteristic vibration frequency in the Arrhenius temperature dependence of the Ga desorption rate, $E_{des}^{\left(3\right)}$ is the activation energy for Ga desorption, T is the temperature in K and $k_{B}$ is the Boltzmann constant. For the ratio of the atomic flux of Ga $I_{Ga}=I_{3}$ over the flux of N_2_ dimers $I_{N_{2}}=I_{5}/2=I_{N}/2$, Equations (4) and (5) then yield our main result:

(6) $$\frac{R_{0}^{2}}{R^{2}}exp\left(-\frac{\Delta E}{k_{B}T}\right)\left[1+\varepsilon exp\left(\frac{\Delta E-E_{des}^{\left(3\right)}}{k_{B}T}\right)\right]<\frac{I_{Ga}}{I_{N_{2}}}<\frac{{\bar{R}}_{0}^{2}}{S/\pi -R^{2}}exp\left(-\frac{\Delta \bar{E}}{k_{B}T}\right)\left[1+\bar{\varepsilon }exp\left(\frac{\Delta \bar{E}-{\bar{E}}_{des}^{\left(3\right)}}{k_{B}T}\right)\right].$$

The parameters are given by:

(7) $$\begin{array}{c} R_{0}^{2}=\frac{1}{\pi }\frac{1}{l_{D}I_{N_{2}}^{3/2}}\frac{{\left(\nu_{des}^{\left(3\right)}\right)}^{2}\sqrt{\nu_{diff}^{\left(5\right)}}}{\nu_{diff}^{\left(3\right)}};\;{\bar{R}}_{0}^{2}=\frac{1}{\pi }\frac{1}{{\bar{l}}_{D}I_{N_{2}}^{3/2}}\frac{{\left({\bar{\nu }}_{des}^{\left(3\right)}\right)}^{2}\sqrt{{\bar{\nu }}_{diff}^{\left(5\right)}}}{{\bar{\nu }}_{diff}^{\left(3\right)}},\\ \Delta E=2E_{des}^{\left(3\right)}+\frac{E_{diff}^{\left(5\right)}}{2}-E_{diff}^{\left(3\right)},\;\Delta \bar{E}=2{\bar{E}}_{des}^{\left(3\right)}+\frac{{\bar{E}}_{diff}^{\left(5\right)}}{2}-{\bar{E}}_{diff}^{\left(3\right)},\\ \varepsilon ={\left[2\sqrt{2}\pi R_{0}^{2}\frac{I_{N_{2}}}{\nu_{des}^{\left(3\right)}}\right]}^{-1},\;\bar{\varepsilon }={\left[2\sqrt{2}\pi {\bar{R}}_{0}^{2}\frac{I_{N_{2}}}{{\bar{\nu }}_{des}^{\left(3\right)}}\right]}^{-1}. \end{array}$$

Let us now analyze the obtained criterion for the optimized SAE growth. First, both ΔE and $\Delta \bar{E}$ values are positive in view of the fact that $E_{des}^{\left(3\right)}>E_{diff}^{\left(3\right)}$ and $E_{diff}^{\left(5\right)}>E_{diff}^{\left(3\right)}$. The first inequality means that Ga adatoms are able to diffuse over a considerable distance before desorption. The second inequality is due to the much higher volatility of N atoms compared to Ga atoms [33]. Therefore, the flux ratio $I_{Ga}/I_{N_{2}}$ corresponding to the SAE region is restricted by the two curves, which increase with temperature, as in the growth diagram shown in Figure 3. Hence, higher temperatures require larger $I_{Ga}/I_{N_{2}}$ ratios, or higher Ga fluxes at a fixed $I_{N_{2}}$ are required to obtain SAE NWs without parasitic nucleation. The upper limit for the Ga/N_2_ flux ratio in Equation (6), $F_{max}={\left(I_{Ga}/I_{N_{2}}\right)}_{max}$, is set by the condition for the absence of parasitic growth. When the Ga flux is too high, nucleation is enabled everywhere on the surface rather than only inside the pinholes. The lower limit, $F_{min}={\left(I_{Ga}/I_{N_{2}}\right)}_{min}$, corresponds to separation of the SAE region from the no-growth region because NWs cannot nucleate inside the pinholes at lower Ga fluxes. Second, the characteristic radii $R_{0}$ and ${\bar{R}}_{0}$ are very large, more than 10^10^ nm, for the plausible vibration frequencies (from 10^10^ to 10^12^ s^−1^ according to [32]). Therefore, the parameters ε and $\bar{\varepsilon }$ must be extremely small due to the presence of the huge factors $\pi R_{0}^{2}/\nu_{des}^{\left(3\right)}$ compared to the modest $I_{N_{2}}$. Neglecting the ε terms, Equation (7) simplifies to:

(8) $$\frac{R_{0}^{2}}{R^{2}}exp\left(-\frac{\Delta E}{k_{B}T}\right)<\frac{I_{Ga}}{I_{N_{2}}}<\frac{{\bar{R}}_{0}^{2}}{S/\pi -R^{2}}exp\left(-\frac{\Delta \bar{E}}{k_{B}T}\right).$$

Third, realization of the SAE mode requires that the radius R and pitch $P=\sqrt{S/\pi }$ for the patterned pinholes. For example, increasing the pitch for a given R increases the mask surface area available for parasitic nucleation. This decreases the upper limit $F_{max}$ due to the larger denominator $S/\pi -R^{2}$, and the SAE mode may not be achieved at all without changing other parameters (for example, increasing the pinhole radius R). 

Figure 3 presents a direct comparison of the model with the data for the GaN NW growth regimes. The curves separating the SAE region were obtained from Equation (6) with the following parameters: $R_{0}=$ 2.52 × 10^14^ nm, ${\bar{R}}_{0}=$ 5.27 × 10^13^ nm, ε= 2 × 10^−19^, $\bar{\varepsilon }=$ 1 × 10^−19^, ΔE= 5.69 eV, $\Delta \bar{E}=$ 5.09 eV, $E_{des}^{\left(3\right)}=$ 2.16 eV and ${\bar{E}}_{des}^{\left(3\right)}=$ 1.72 eV. While no literature data are available for the first four parameters, the obtained values for the activation energies seem plausible and correlate with previously published results [32]. In particular, the 2.16 eV activation energy for Ga desorption on Si(111) was smaller than 2.75 eV on the GaN m-plane and within the typical range of 2.0 eV to 5.1 eV on the GaN c-plane, according to the data from [32]. The activation energy for Ga desorption on Si(111) appeared to be larger than on amorphous SiO_x_ (1.72 eV), which is also reasonable. Overall, the quantitative correlation of the model with the data shown in Figure 3 was very good. 

**Figure 4:**
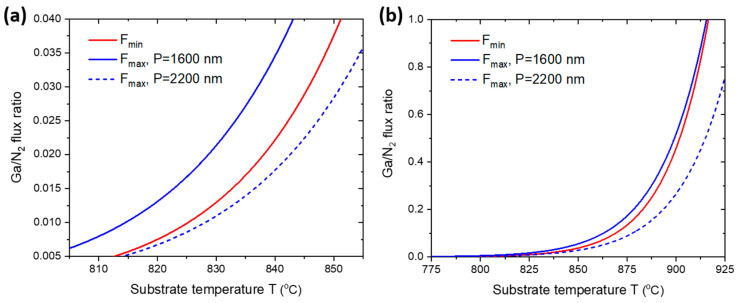
(**a**) The maximum ($F_{max}$) and minimum ($F_{min}$) Ga/N_2_ flux ratios separating the SAE region with the same range as in Figure 3. The solid curves are the same as in Figure 3. The dashed curve is the maximum Ga/N_2_ flux ratio with the same parameters but with an increased pitch of 2000 nm instead of 1600 nm. Increasing the pitch led to the disappearance of the SAE region in the investigated range of temperatures and Ga/N_2_ flux ratios. (**b**) The absence of the SAE zone with a larger range of temperatures and Ga/N_2_ flux ratios.

Figure 4 illustrates the effect of geometry on the SAE region for the same parameters as above but with the pitch of the pinholes increased from 1600 nm to 2200 nm. In both figures, the maximum Ga/N_2_ flux ratio is lower than the minimum. Therefore, SAE of GaN NWs cannot be realized for pinholes with a larger pitch without changing other growth parameters, as discussed above. Therefore, SAE of GaN NWs on patterned SiO_x_/Si(111) substrates with 100% growth selectivity requires careful optimization of the MBE parameters along with the geometry of the growth template.

### 3.3. Optical Studies

**Figure 5:**
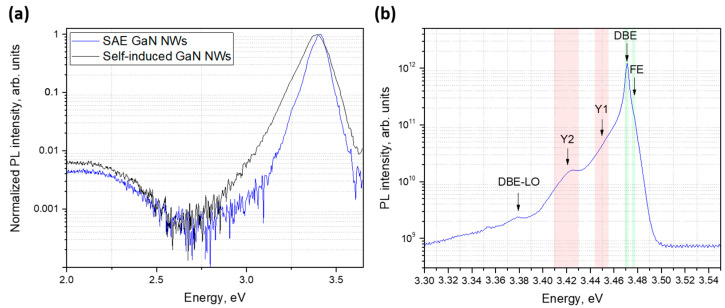
(**a**) RT PL spectra from the self-induced (black line) and SAE (blue line) GaN NWs. (**b**) Low-temperature (6 K) PL spectrum from the SAE GaN NWs, where the five peaks corresponding to the FE, DBE, defect-related Y_1_ and Y_2_ and DBE-LO transitions are indicated. The PL intensity is given in a logarithmic scale.

The optical studies were carried out for the SAE GaN NWs grown at 830 °C and a Ga flux corresponding to 3 × 10^−7^ Torr BEP (see Figure 2b). First, room temperature photoluminescence (RT PL) was measured and compared to the irregular self-induced GaN NWs grown on bare Si(111) using PA-MBE under similar conditions. The black and red lines in Figure 5a show the RT PL spectra of the self-induced GaN NWs and SAE GaN NWs, respectively. Both spectra exhibited the PL peak at about 3.41 eV, corresponding to near-band-edge (NBE) optical transitions in GaN, and a defect-related PL band with a maximum at about 2.15 eV [34]. The intensity of defect-related PL peaks was two orders of magnitude lower than the NBE peaks for both samples. The full-width at half-maxima (FWHM) of the NBE PL peaks from self-induced and SAE GaN NWs were 0.38 and 0.26 eV, respectively. However, the ratio of the defect-related PL intensity over the NBE intensity and the FWHM of the NBE peak at room temperature do not directly confirm the high structural quality of GaN [34,35]. To further elaborate this, Figure 5b shows the low-temperature PL spectrum of the SAE GaN NWs, measured at 6 K. This PL spectrum exhibited 5 emission peaks corresponding to the donor-bound exciton (DBE), free exciton (FE), defect-related Y_1_ and Y_2_ signals, as well as a phonon replica (DBE-LO) [34,36].

According to Figure 5b, the DBE line at 3.471 eV was the strongest PL line, with a shoulder FE line at 3.477 eV. The FWHM of the DBE peak was 3.6 meV. The DBE line position clearly matches with strain-free GaN NWs (see [37] and references therein). The FWHM values are very close to GaN nanocolumns grown on AlN/Al_2_O_3_ and AlN/Si(111) substrates (FWHM ~ 1.26 meV obtained at unshown excitation density) [38]. To the best of our knowledge, the FWHM value given in [38] is still the record for arrays of GaN NWs [37,38,39,40]. The Y_1_ and Y_2_ signals are often not observed in GaN layers but are typical for GaN NWs [6,37,41]. The Y_1_ signal at 3.443–3.455 eV can originate from two-photon satellites [34,36], prismatic inversion domain boundaries [42], or donor-bound excitons close to the nanowire surface [43]. To shed more light on the nature of the Y1 signal, the micro-PL measurements of single NWs are required. The Y_2_ signal at 3.41–3.43 eV has been attributed to excitons bound to structural defects, such as stacking faults from coalesced NWs [37] and the bottom interface of NWs [39,44]. It is noteworthy that the ratios of DBE over the Y_1_ and Y_2_ intensities were 1/25 and 1/82, respectively, which is comparable with the previously published values for high-crystal-quality GaN NWs grown on diamond [39], Al_2_O_3_ and Si(111) substrates with an AlN buffer layer [38]. All these features confirm a high crystal quality for the grown SAE GaN NWs. 

## 4. Conclusions

In summary, SAE GaN NWs were grown using PA-MBE on SiO_x_/Si(111) substrates using microsphere lithography to pattern regular arrays of pinholes in a silica mask. The SAE, no-growth and parasitic-growth regions were separated on the temperature–Ga/N_2_ flux ratio map. The SAE domain appeared to be rather narrow, and it was highly sensitive not only to temperature and Ga flux but also to the radius and pitch of the patterned pinholes according to the model. High crystal quality in the SAE GaN NWs was confirmed using PL studies. We now plan to extend the model by accounting for the possible Ga diffusion flux into or from the pinoles and other factors that were neglected in the simplified approach. We also plan to study in detail the role of pinhole array geometry in growth selectivity. Overall, these findings should be useful for obtaining regular arrays of GaN NWs without any parasitic growth on a mask surface and can be extended to other material systems and growth techniques. Therefore, our work opens up new prospects for the creation of LEDs and sources of single and entangled photons based on III-N compounds. 

## Data Availability

Not applicable.
